# Donepezil as a new therapeutic potential in KCNQ2- and KCNQ3-related autism

**DOI:** 10.3389/fncel.2024.1380442

**Published:** 2024-08-08

**Authors:** Andreea Nissenkorn, Lior Bar, Ariel Ben-Bassat, Lynn Rothstein, Hoda Abdelrahim, Riki Sokol, Lidia V. Gabis, Bernard Attali

**Affiliations:** ^1^Pediatric Neurology Unit, Edith Wolfson Medical Center, Holon, Israel; ^2^Magen National Center for Rare Disorders, Edith Wolfson Medical Center, Holon, Israel; ^3^Department of Pediatric, School of Medicine, Tel-Aviv University, Tel Aviv, Israel; ^4^Department of Electrophysiology, School of Medicine, Tel Aviv University, Tel Aviv, Israel

**Keywords:** personalized medicine, gain-of-function, donepezil, KV7 channels, voltage-gated channels, KCNQ2, KCNQ3

## Abstract

**Introduction:**

The *KCNQ2/KCNQ3* genes encode the voltage-gated K channel underlying the neuronal M-current, regulating neuronal excitability. Loss-of-function (LoF) variants cause neonatal epilepsy, treatable with the M-current-opener retigabine, which is no longer marketed due to side effects. Gain-of-function (GoF) variants cause developmental encephalopathy and autism that could be amenable to M-current, but such therapies are not clinically available. In this translational project, we investigated whether donepezil, a cholinergic drug used in Alzheimer’s, suppresses M currents *in vitro* and improves cognitive symptoms in patients with GoF variants.

**Methods:**

(1) The effect of 1 μM donepezil on the amplitude of the M-current was measured in excitatory and inhibitory neurons of mouse primary cultured hippocampal cells. M-current was measured using the standard deactivation protocol (holding at 0 mV and deactivation at −60 mV) in the voltage-clamp configuration of the whole-cell patch clamp technique. The impact of donepezil was also examined on the spontaneous firing activity of hippocampal neurons in the current-clamp configuration. (2) Four children with autism, aged 2.5–8 years, with the following GoF variants were enrolled: *KCNQ2 (p. Arg144Gln)* and *KCNQ 3 (p.Arg227Gln, p.Arg230Cys)*. Patients were treated off-label with donepezil 2.5–5 mg/d for 12 months and assessed with: clinical Global Impression of Change (CGI-c), Childhood Autism Rating Scale 2 (CARS-2), Adaptive Behavior Assessment System-II (ABAS-II), and Child Development Inventory (CDI).

**Results:**

(1) Application of donepezil for at least 6 min produced a significant inhibition of the M-current with an IC50 of 0.4 μM. At 1 μM, donepezil reduced by 67% the M-current density of excitatory neurons (2.4 ± 0.46 vs. 0.89 ± 0.15 pA/pF, *p* < 0.05^*^). In inhibitory neurons, application of 1 μM donepezil produced a lesser inhibition of 59% of the M-current density (1.39 ± 0.43 vs. 0.57 ± 0.21, *p* > 0.05). Donepezil (1 μM) potently increased by 2.6-fold the spontaneous firing frequency, which was prevented by the muscarinic receptor antagonist atropine (10 μM). (2) The CARS-2 decreased by 3.8 ± 4.9 points (*p* > 0.05), but in two patients with *KCNQ3* variants, the improvement was over the 4.5 clinically relevant threshold. The global clinical change was also clinically significant in these patients (CGI-c = 1). The CDI increased by 65% (*p* < 0.05^*^), while the ABAS-II remained unchanged.

**Discussion:**

Donepezil should be repurposed as a novel alternative treatment for GoF variants in *KCNQ2/KCNQ3* encephalopathy.

## 1 Introduction

Neuronal M channels, belonging to the voltage-gated potassium channel Kv7 family, have emerged as critical players in epilepsy and neurodevelopmental disorders (Marrion, 1997; Cooper and Jan, 2003; Wladyka and Kunze, 2006; Cooper, 2012). The term “M-current” was used initially to describe a K current that was inhibited by a muscarinic agonist (Brown and Adams, 1980). This current is generated by the opening of voltage-gated potassium channels Kv7, producing a non-inactivating subthreshold outward rectifying K^+^ current, which regulates neuronal excitability (Marrion, 1997; Jentsch, 2000; Cooper and Jan, 2003; Delmas and Brown, 2005). The M channels are assembled as heterotetramers of Kv7.2, Kv7.3, and Kv7.5 subunits, and play an important role in epilepsy and neurodevelopmental disorders (Kv7.2 and Kv7.3 subunits) (Marrion, 1997; Cooper and Jan, 2003; Wladyka and Kunze, 2006; Cooper, 2012). The term KCNQ refers to the respective genes encoding the Kv7 channel proteins (Wang et al., 1998). Pathogenic variants in the *KCNQ2* (HGNC:6296) and *KCNQ3* (HGNC:6297) genes were initially associated with self-limited neonatal epilepsy (previously known as benign neonatal epilepsy) (Biervert et al., 1998; Singh et al., 2003; Zuberi et al., 2022) and later with early-onset developmental and epileptic encephalopathy with burst suppression pattern on EEG (previously known as Ohtahara syndrome) (Weckhuysen et al., 2012; Zuberi et al., 2022). This phenotype is caused by pathogenic variants causing LoF, and electrophysiological studies have shown a correlation between the extent of dysfunction and clinical severity (Hunter et al., 2006; Miceli et al., 2009; Lee et al., 2017). Kv7 channel openers such as retigabine (also named ezogabine) that were developed as antiseizure medications were shown to be effective as targeted therapy in patients with LoF variants in *KCNQ2* (Millichap et al., 2016; Nissenkorn et al., 2021). However, this medication was lately withdrawn from marketing due to the potential side effects such as retinal toxicity and blue-colored skin discoloration.^1^

Recently, GoF variants in the *KCNQ2* gene were described, causing a distinct phenotype with profound developmental delay, neonatal non-epileptic myoclonus, and later onset epilepsy such as infantile spasms (Miceli et al., 2015; Millichap et al., 2017; Mulkey et al., 2017; Miceli et al., 2022). In addition, GoF variants in the *KCNQ3* gene were described, causing a phenotype with autism and epileptic discharges on EEG during sleep, but no clinical seizures (Miceli et al., 2015; Sands et al., 2019). Unfortunately, these patients are not amenable to personalized medicine since there are no Kv7 channel blockers clinically available.

However, patch clamp analysis studies showed that linopirdine, a cholinergic compound not in clinical use, is a state-dependent inhibitor of Kv7 channels, which suppresses the M currents (Greene et al., 2017). Along this line, it is well known that the activation of the Gq-coupled muscarinic acetylcholine receptor activates a phospholipase C β, which leads to the hydrolysis of membrane phosphatidylinositol-4,5-bisphosphate (PIP2) that indirectly causes the closure of the M channels because their gate opening depends on the presence of PIP2 (Delmas and Brown, 2005; Gamper and Shapiro, 2007a,b).

In this translational project, we investigated whether the cholinergic drug donepezil, an inhibitor of the brain acetylcholinesterase widely used in Alzheimer’s disease (Bryson and Benfield, 1997), can act as an indirect Kv7 channel blocker *in vitro* by increasing the cholinergic tonus and thus improving cognitive symptoms in patients with GoF variants in the *KCNQ2* and *KCNQ3* genes.

## 2 Materials and methods

### 2.1 Laboratory studies

#### 2.1.1 Animals

Balb/c mice of either sex were used for generating the primary cultures of hippocampal neurons. All experimental protocols conformed to the guidelines of the Institutional Animal Care and Use Committee of Tel Aviv University, Israel, and to the guidelines of the NIH (animal welfare authorization number 01-16-012).

#### 2.1.2 Drugs

Tetrodotoxin citrate (TTX) (Alomone; Cat.No. T-550), 1(S),9(R)-(−)-bicuculline methiodide (Sigma; Cat.No. 14343), picrotoxin (Sigma; Cat.No. P1675), NBQX hydrate (Sigma; Cat.No. N171), AP5-DL-2-amino-5-phosphonopentanoic acid (Sigma; Cat.No. A5282), and atropine sulfate salt monohydrate (Sigma, A0257).

#### 2.1.3 Primary cultures of hippocampal neurons

Hippocampi were dissected out from neonate Balb/c mice brains (0–1 day old). Hippocampi were washed three times in an HBSS-based solution containing: 4 mM NaHCO_3_, 5 mM HEPES, and Hank’s balanced salt solution (Sigma), pH adjusted to 7.3–7.4 at 4°C. Tissues were digested in a solution including: 137 mM NaCl, 5 mM KCl, 7 mM Na_2_HPO_4_, 25 mM HEPES, 4.45 mg/mL of trypsin type XI (Sigma), and 1,614 U/mL of DNase type IV (Sigma), pH adjusted to 7.2 at 4°C. Hippocampal tissues were incubated for 10 min at 37°C and washed once with 5 mL of HBSS/20% fetal bovine serum (FBS) and once with HBSS. The cells were dissociated in a HBSS solution including 13.15 mM MgSO_4_ and 1772 U/mL of DNase type IV (Sigma). Next, the cells were mechanically triturated with fire-polished Pasteur pipettes. HBSS/20% FBS was added to the dissociated cells, and the mixture was centrifuged at 1000 × g, at 4°C for 10 min. The supernatant was discarded and a plating medium including MEM (Gibco), 24.7 mM glucose, 0.089 mg/mL of transferrin (Calbiochem), GlutaMAX (Gibco), 0.75 U/mL of insulin (Sigma), 10% FBS (Biological Industries), and SM1 (StemCell NeuroCult neuronal supplement) was added to the pellet. The cells were resuspended in the plating medium with fire-polished Pasteur pipette and viable cells were counted. A measure of 0.5 mL of the cell suspension was added to glass coverslips coated with Matrigel (Corning) in a 24-wells plate at a density of ~180,000 cells per well. Two days after plating, 0.5 mL of feeding medium [MEM, 26.92 mM glucose, 0.097 mg/mL of transferrin, GlutaMAX, SM1, and 3 μM cytosine arabinoside (Ara-C) (Sigma)] was added to each well. Twice a week, half of the medium was removed from the wells and replaced with the same volume of feeding medium.

#### 2.1.4 Recombinant AAV-Dlx-mCherry plasmid and infection

To identify GABAergic neurons, we infected hippocampal cultures with a recombinant virus derived from an AAV-viral vector driving the expression of the fluorescent protein mCherry under the control of the specific GABAergic hDlx promoter. Recombinant AAV-virus-Dlx-mCherry plasmid was prepared by inserting the hDlx promoter sequence (541 bp) upstream of the coding sequence in the backbone of the pAAV2-mCherry plasmid. The Dlx promoter was shown to restrict reporter expression *in vivo* to all GABAergic interneurons in the forebrain, including the hippocampus, as well as in cultured neurons *in vitro* (Dimidschstein et al., 2016; Lezmy et al., 2020). The recombinant AAV2-virus-Dlx-mCherry was produced using standard production methods in HEK 293 cells in the recombinant virus production facility of Tel Aviv University. All batches produced were in the range of 10^9^–10^10^ viral particles per ml. Infections of hippocampal cultures were performed at 6 DIV, and recording was carried out at 14–16 DIV.

#### 2.1.5 Patch clamp electrophysiology

Patch clamp was performed in the whole-cell configuration. Signals were filtered at 4 kHz and digitized at 10 kHz. All signals were amplified using the MultiClamp 700B (Molecular Devices). M currents were recorded in the voltage-clamp configuration where the extracellular solution contained 140 mM NaCl, 2.5 mM KCl, 5 mM HEPES, 5 mM glucose, 1.2 mM MgCl_2_, and 1.8 mM CaCl_2_ (pH was adjusted to 7.4 with NaOH, osmolarity~315 mOsm); 1 μM TTX and 0.2 mM 4-aminopyridine were added to the extracellular solution to block voltage-gated Na^+^ currents and IA K^+^ currents, respectively. Synaptic blockers were added to the extracellular solution to prevent spontaneous spikes: 30 μM picrotoxin, 10 μM Bicuculline, 10 μM NBQX, and 10 μM AP5. The intracellular solution contained: 130 mM K-gluconate, 6 mM KCl, 2 mM Na_2_ATP, 10 mM HEPES, 1.1 mM EGTA, and 0.1 mM CaCl_2_ (pH adjusted to 7.2.5 with KOH, osmolarity~300 mOsm). All electrophysiological experiments were performed at room temperature. The M-current was measured by the standard deactivation protocol as previously described (Bar et al., 2022). Neurons were held at −60 mV. A step to −20 mV was then given for 6 s, to open M currents and remove residual inactivating voltage-dependent currents. Then, the voltage was brought back to −60 mV for 4 s to close M currents, followed by another −20 mV step for 2 s. After offline leak subtraction, the M-current was calculated by the amplitude of the tail that was corrected by the capacitance of the cell and was expressed as pA/pF. In the current-clamp configuration, the input resistance was measured by a 400 ms hyperpolarizing current injection of −100 pA. Donepezil hydrochloride (Sigma) was dissolved as a 10 mM stock solution in distilled water and was then diluted in the extracellular recording solution. The effects of donepezil were examined on the amplitude of the tail of the M-current measured separately in excitatory and inhibitory neurons of primary cultured hippocampal cells (14–16 d *in vitro*) as previously detailed (Bar et al., 2022). For recordings in the current-clamp configuration, the extracellular solution contained 160 mM NaCl, 2.5 mM KCl, 10 mM HEPES, 10 mM glucose, 1.2 mM Mg^2+^, and 1.8 mM Ca^2+^ (pH was adjusted to 7.3 with NaOH; osmolarity ≈315 mOsm). The intracellular solution contained: 135 mM KCl, 1 mM KATP, 1 mM MgATP, 2 mM EGTA, 1.1 mM CaCl_2_, 10 mM HEPES, and 5 mM glucose (pH adjusted to 7.3 with KOH, osmolarity≈300 mOsm).

All graphs were built with Prism 9.0 (GraphPad). Error bars represent the standard error of the mean (SEM). Statistical comparisons between untreated and donepezil-treated cells were performed using two-tailed Wilcoxon matched-pairs signed rank tests.

### 2.2 Clinical intervention

#### 2.2.1 Population

Patients with *KCNQ2-* or *KCNQ3*-related developmental encephalopathy were enrolled if they fulfilled the following criteria:

1. Variants occurring at the following “hot spots,” which have been proven to cause gain in channel function in previous electrophysiological studies: *KCNQ2*, Arg 144 (Miceli et al., 2015, 2022), *KCNQ2*, Arg198 (Millichap et al., 2017), *KCNQ2,* Arg 201 (Miceli et al., 2015; Mulkey et al., 2017), *KCNQ3,* Arg227 (Sands et al., 2019), *KCNQ3*, and Arg230 (Miceli et al., 2015; Sands et al., 2019).
2. Phenotype was consistent with one of the *KCNQ2* or *KCNQ3* GoF phenotypes described in the literature: autism and intellectual disability (Sands et al., 2019), autism and infantile and childhood epilepsy (Miceli et al., 2022), infantile spasms or early onset epileptic encephalopathy (Miceli et al., 2015; Millichap et al., 2017), and neonatal encephalopathy with non-epileptic myoclonus (Mulkey et al., 2017).

#### 2.2.2 Treatment

Treatment with donepezil was administered off-label, according to compassionate use approval, separate for each patient, after the parents signed an informed consent. The dosage administered was 5 mg once a day for children over 4 years of age and 2.5 mg once a day for children under 4 years of age. The dosage was extrapolated from previous studies in which donepezil was used in children with autism (Chez et al., 2003; Gabis et al., 2019). Tablets were either swallowed or crushed and administered with water. The treatment was started at half dosage (2.5 and 1.25 mg, respectively) and tapered up to full dosage after 2 weeks. Dosage was adjusted according to side effects at 4 weeks, 12 weeks, and 6-month visits.

#### 2.2.3 Outcome measures

The following cognitive and behavioral measures were assessed at baseline and at 6 months and 12 months visits: CARS-2, ABAS-II, and CDI. The CARS-2 is a clinical rating scale designed to identify children with autism spectrum disorder and determine symptom severity (Schopler et al., 2010). The cutoff point for autism is 28 points (Schopler et al., 2010). A 4.5-point decrease in CARS-2 is suggested in the literature as the threshold for clinically relevant changes in autistic features (Jurek et al., 2022). The ABAS-II^**^ is a comprehensive norm-referenced assessment of the adaptive skills of individuals that quantifies daily life skills and is used in interventional studies as an outcome measure (Oakland, 2008). The ABAS-II includes 10 skills that are grouped into three broad domains: conceptual, social, and practical (Oakland, 2008). The CDI is a caretaker-reported tool used to assess developmental milestones in children. The questionnaire includes various domains of development, including social, self-help, motor, language, letter, and number skills (Ireton and Glascoe, 1995). For ABAS-II and CDI, we arbitrarily considered a 20% increase in score as a clinically significant change. In addition, caregivers were also administered the CGI-c at 6-month and 12-months follow-up visits. The CGI is a widely used scale that rates overall disease severity (CGI-s) or change in severity over time (CGI-c). The CGI-c is an accepted outcome measure for interventional trials in neurodevelopmental disorders. It is graded from 1 (very much improved) to 7 (very much worsened). We considered a score of 1 (very much improved) as a clinically prominent improvement. After the 12-month evaluation, the results were discussed with the caregivers, and they opted accordingly to continue or withdraw from the study.

#### 2.2.4 Statistical analysis

Descriptive statistics were used to tabulate the parameters. We compared numeric parameters between multiple related groups (before treatment, 6 months after intervention, and 12 months after intervention) using the Friedman test. Significance values have been adjusted by the Bonferroni correction for multiple tests. Data were analyzed using the SPSS software (IBM^®^SPSS^®^ version 27).

This publication was approved by the Local Helsinki Committee at the Wolfson Medical Center - IRB#0150-23-WOMC.

## 3 Results

### 3.1 Laboratory study

**Figure 1:**
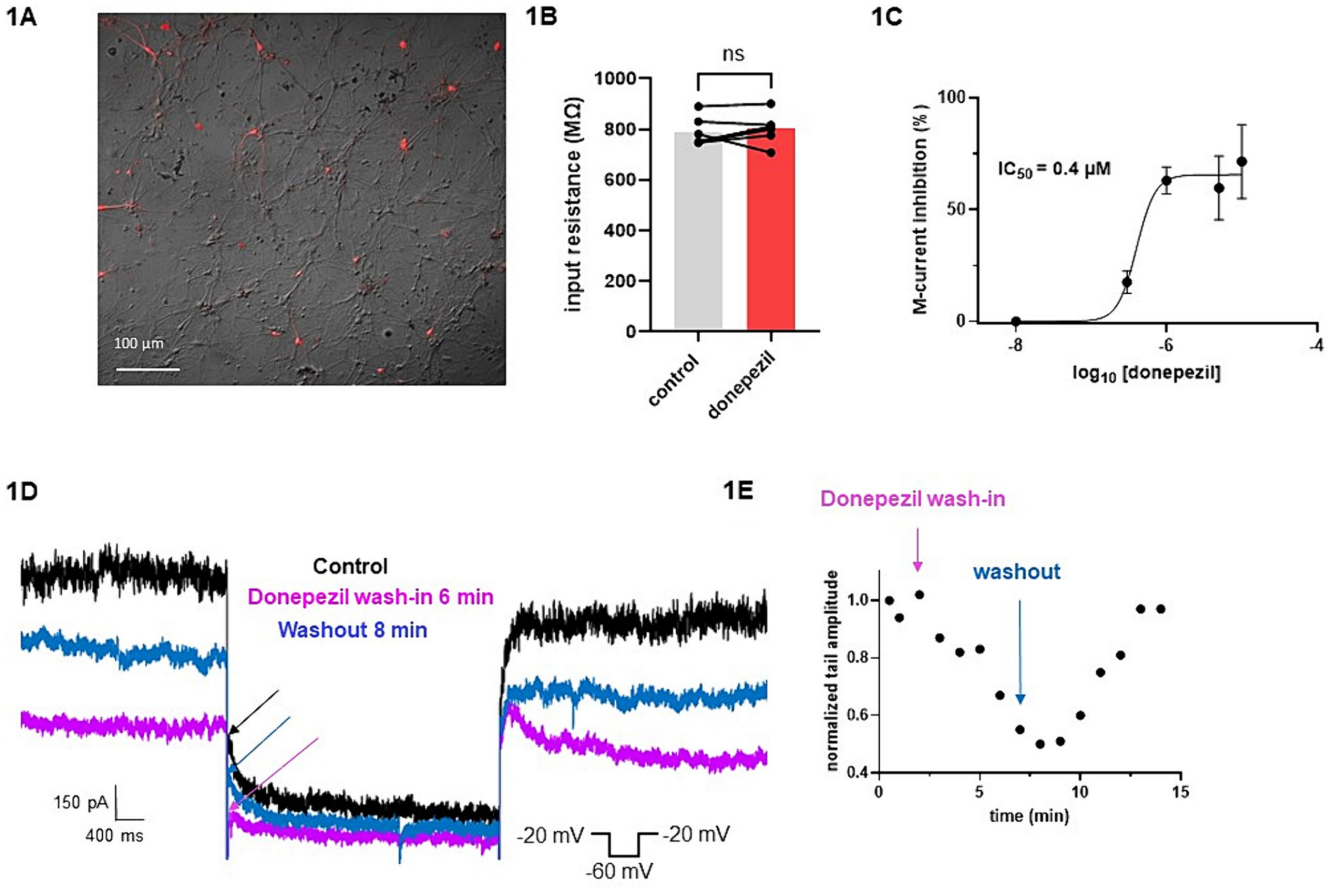
Effects of donepezil on input resistance and M-current density: wash-in and washout. **(A)** Cultured hippocampal neurons are shown in bright field, superimposed with mCherry fluorescence discriminating GABAergic inhibitory neurons (red fluorescent neurons) from excitatory neurons (non-fluorescent neurons). **(B)** Application of donepezil to excitatory neurons did not significantly affect their input resistance (785 ± 21 MΩ and 803 ± 22 MΩ for control and donepezil-treated neurons, respectively; *n* = 7, two-tailed Wilcoxon matched-pairs signed rank test, *p* = 0.4688). **(C)** Application of donepezil to excitatory neurons produced a significant reduction of the M-current with an IC_50_ of 0.4 μM. **(D)** Representative traces showing donepezil effects on M-current measured in an excitatory neuron. The arrows show the amplitude of the tail (peak tail) that was measured at the beginning of the step at −60 mV. The donepezil wash-in reached completion within 6–7 min, while its washout was complete by 8–9 min. **(E)** Typical time-course of donepezil inhibitory action on the M-current measured in an excitatory neuron.

**Figure 2:**
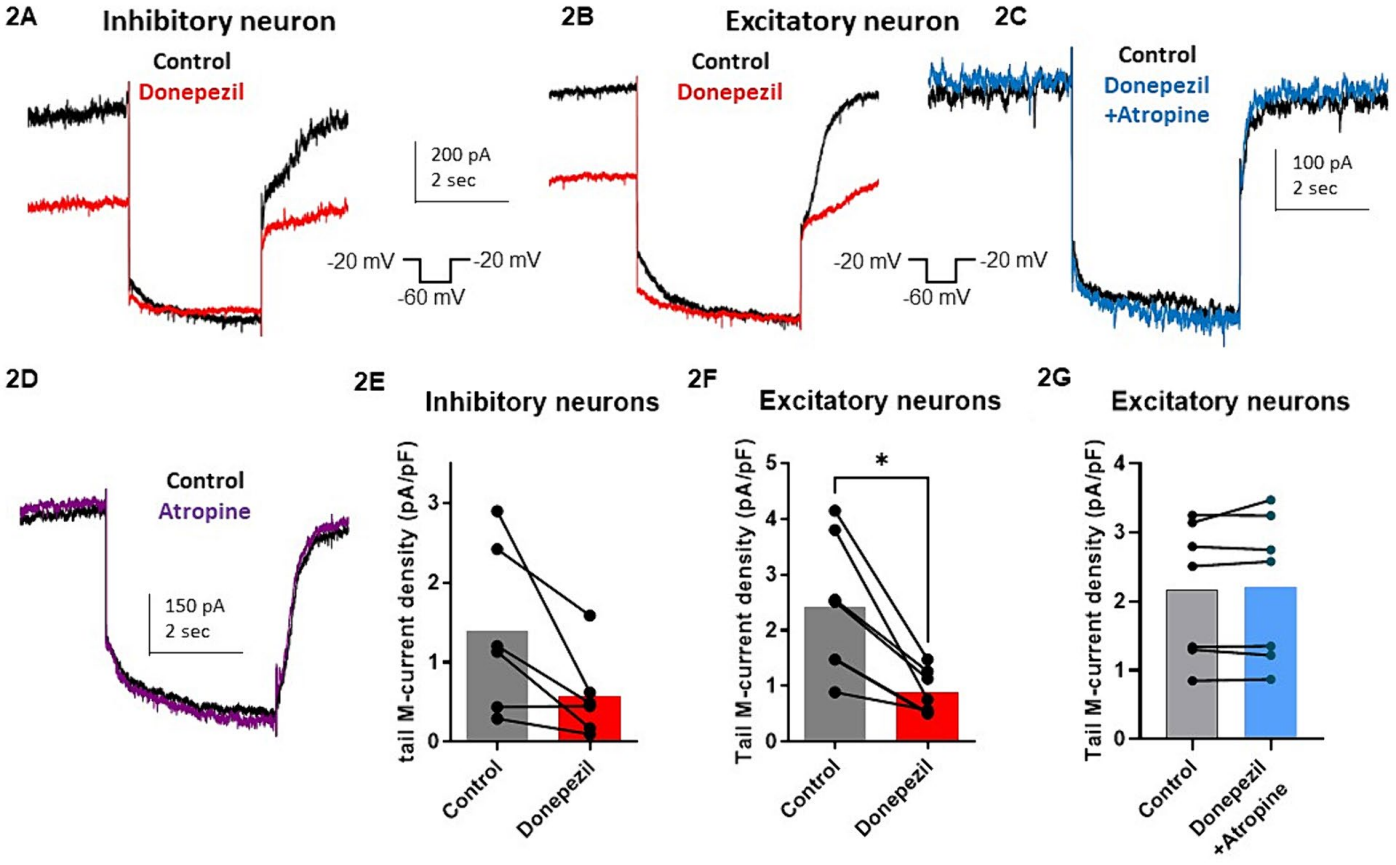
Comparative effect of donepezil (1 μM) on M currents measured in hippocampal excitatory and inhibitory neurons. **(A,B)** Representative traces of the M-current measured by the standard deactivation protocol in an inhibitory neuron **(A)** and an excitatory neuron **(B,C)**, control traces of the tail current are depicted in black and donepezil traces in red (left panel) while donepezil + atropine trace in blue. **(D)** The trace shows that atropine alone does not affect significantly the current. **(E)** M-current density in inhibitory neurons is decreased by 59% after application of 1 μM donepezil (from 1.39 ± 0.43 pA/pF to 0.57 ± 0.22 pA/pF, *n* = 6, two-tailed Wilcoxon matched-pairs signed rank test, *p* > 0.05). **(F)** Application of 1 μM donepezil produced a significant inhibition of 67% of the M-current density in excitatory neurons (from 2.40 ± 0.46 pA/pF to 0.88 ± 0.14 pA/pF, *n* = 7, two-tailed Wilcoxon matched-pairs signed rank test, *p* < 0.05). **(G)** Atropine (10 μM) prevented the M-current density inhibition produced by donepezil (*n* = 7; 2.2 ± 0.4 pA/pF and 2.2 ± 0.4 pA/pF for control and donepezil + atropine, treated neurons respectively; two-tailed Wilcoxon matched-pairs signed rank test, *p* = 0.6875).

**Figure 3:**
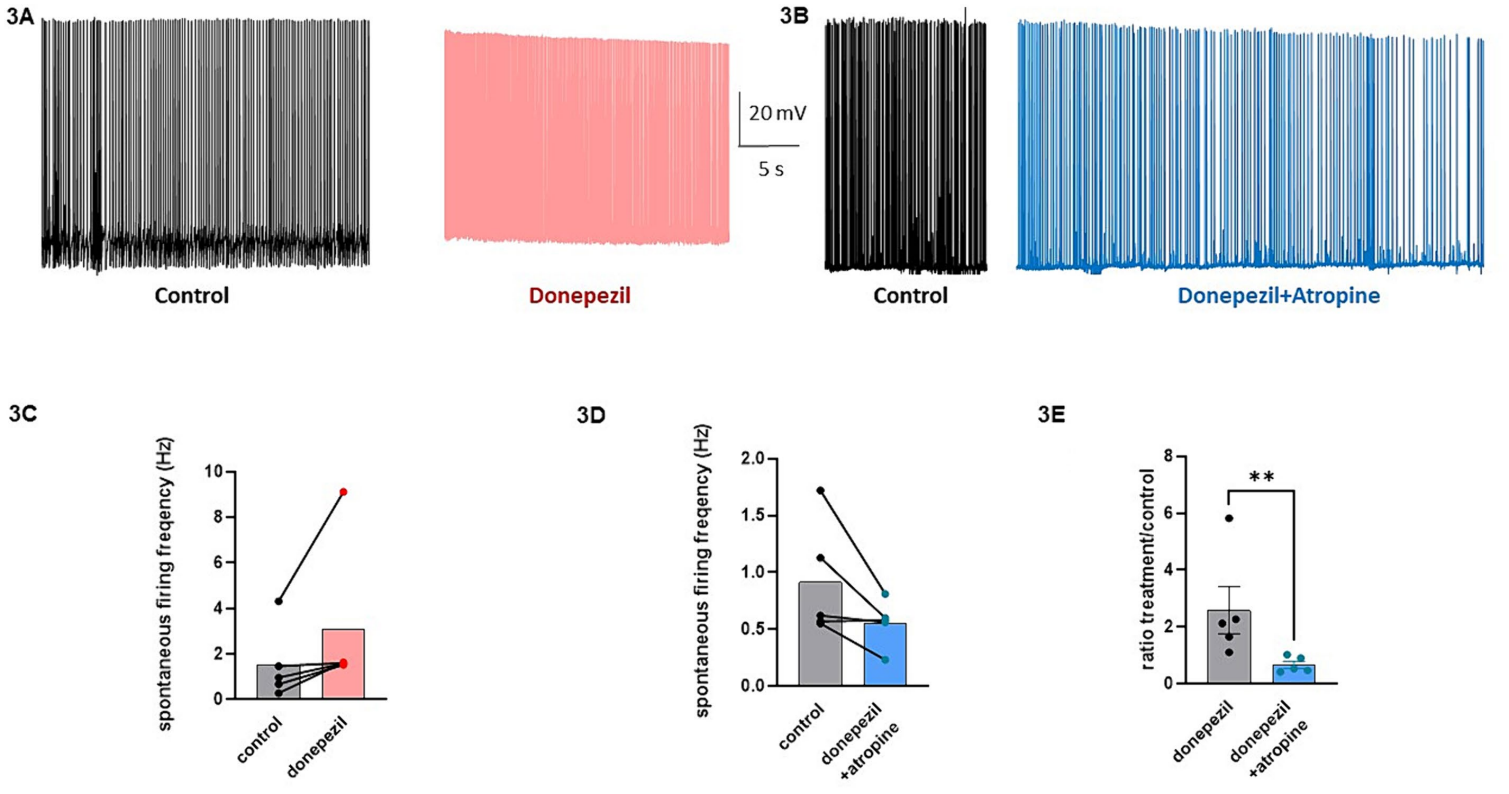
Effects of donepezil on the spontaneous firing frequency of excitatory hippocampal neurons. **(A)** Representative trace of the spontaneous firing of a neuron before (black) and following 5 min application of 1 μM donezepil (pink). **(B)** Representative trace of the spontaneous firing of a neuron before (black) and following 5 min application of 1 μM donezepil + 10 μM atropine (blue). **(C)** Donezepil potently increased the spontaneous firing frequency (*n* = 5, 1.5 ± 0.7 Hz and 3.1 ± 1.5 Hz for control and donepezil-treated neurons, respectively). **(D)** Atropine (10 μM) treatment prevented the increased network firing produced by donepezil and even consistently reduced the spiking frequency (*n* = 5, with 0.9 ± 0.2 Hz and 0.5 ± 0.1 Hz for control and donepezil + atropine treated neurons, respectively). **(E)** Comparing the donepezil and donepezil + atropine treated neurons, resulted in a significantly higher frequency ratio of treatment vs. control (*n* = 5, ratio of 2.6 ± 0.8 and 0.7 ± 0.1 for donepezil and donepezil + atropine treatment, respectively; two-tailed Mann–Whitney test, ^**^p = 0.0079).

The effect of donepezil was first examined on the input resistance and the M-current density of excitatory neurons in primary cultured hippocampal cells (14–16 d *in vitro*), To distinguish between GABAergic inhibitory neurons and excitatory neurons, we infected hippocampal cultures with a recombinant virus derived from an AAV-viral vector driving the expression of the fluorescent protein mCherry under the control of the specific GABAergic hDlx promoter. Thus, excitatory neurons appear as non-fluorescent pyramidal-like cells, while inhibitory neurons appear as red fluorescent cells (Figure 1A). Application of donepezil to excitatory neurons did not significantly affect their input resistance (785 ± 21 MΩ and 803 ± 22 MΩ for control and donepezil-treated neurons, respectively; *n* = 7 two-tailed Wilcoxon matched-pairs signed rank test, *p* = 0.4688) (Figure 1B). The M-current density was measured by the standard deactivation protocol (holding at −20 mV and deactivation at −60 mV) in the whole-cell configuration of the patch clamp technique and was determined by the amplitude of the tail (see the arrows shown in Figure 1D) and corrected by the capacitance of the cell. Application of donepezil to excitatory neurons produced a significant reduction of the M-current with an IC_50_ of 0.4 μM and reached saturating inhibition by 1 μM concentration (Figure 1C). The time-course of donepezil inhibitory action on the M-current was slow. Donepezil wash-in reached completion within 6–7 min, while its washout was complete by 8–9 min (Figures 1D,E). We next compared the donepezil action in excitatory and inhibitory neurons. Application of 1 μM donepezil produced a significant inhibition of 67% of the M-current density in excitatory neurons (from 2.40 ± 0.46 pA/pF to 0.88 ± 0.14 pA/pF, *n* = 7, two-tailed Wilcoxon matched-pairs signed rank test, *p* < 0.05) (Figures 2B,F). Importantly, the muscarinic receptor antagonist atropine (10 μM) prevented the M-current density inhibition produced by donepezil, suggesting that the muscarinic receptor signaling was involved in the donepezil effect (Figures 2C,G; *n* = 7; 2.2 ± 0.4 pA/pF and 2.2 ± 0.4 pA/pF for control and donepezil + atropine, treated neurons, respectively; two-tailed Wilcoxon matched-pairs signed rank test, *p* = 0.6875). Atropine alone did not significantly affect the M-current (Figure 2D). In inhibitory neurons, application of 1 μM donepezil produced a slightly weaker inhibition of 59% of the M-current density that did not reach statistical significance (from 1.39 ± 0.43 pA/pF to 0.57 ± 0.22 pA/pF, *n* = 6, two-tailed Wilcoxon matched-pairs signed rank test, *p* > 0.05) (Figures 2A,E). To examine the impact of donepezil inhibition of the M-current density on hippocampal network excitability, we measured the spontaneous firing frequency in the absence and presence of donepezil (1 μM) (Figures 3A,C) on excitatory hippocampal neurons. In agreement with the M-current inhibition, the results show that donepezil depolarized the membrane potential and potently increased the spontaneous firing frequency (Figures 3A,C; *n* = 5, 1.5 ± 0.7 and 3.1 ± 1.5 Hz for control and donepezil-treated neurons, respectively). Noticeably, atropine (10 μM) treatment prevented the increased network firing produced by donepezil and even consistently reduced the spiking frequency, suggesting the existence of an endogenous tonic cholinergic excitability in the cultured hippocampal network (Figures 3B,D; *n* = 5, with 0.9 ± 0.2 Hz and 0.5 ± 0.1 Hz for control and donepezil + atropine-treated neurons, respectively). Comparing the donepezil and donepezil + atropine-treated neurons resulted in a significantly higher frequency ratio of treatment vs. control (Figure 3E; *n* = 5, ratio of 2.6 ± 0.8 and 0.7 ± 0.1 for donepezil and donepezil + atropine treatment, respectively; two-tailed Mann–Whitney test, ^**^p = 0.0079).

### 3.2 Clinical intervention

#### 3.2.1 Population

Four patients, bearing three GoF variants, aged 2.5–8 years of age (median 4 years, mean 4.6 ± 2.3), three females and one male, were enrolled (Table 1).

**Table 1:** Phenotypic characterization of the study group.

Patient	1	2	3	4
Pathogenic variant	KCNQ3, p. ArgR227Gln	KCNQ3, p. Arg230Cys	KCNQ3, p. Arg230Cys	KCNQ2, p. Arg144Gln
Age (years)	3.5	8	2.5	4.5
Gender	Female	Male	Female	Female
Phenotype	ASD/DE	ASD/DE	ASD/DE	ASD/DE
Seizures	−	−	−	+
Sleep EEG	Multifocal spike and wave	Normal	Multifocal spike and wave	Multifocal spike and wave

ASD, autism spectrum disorder; DE, developmental encephalopathy.

**Figure 4:**
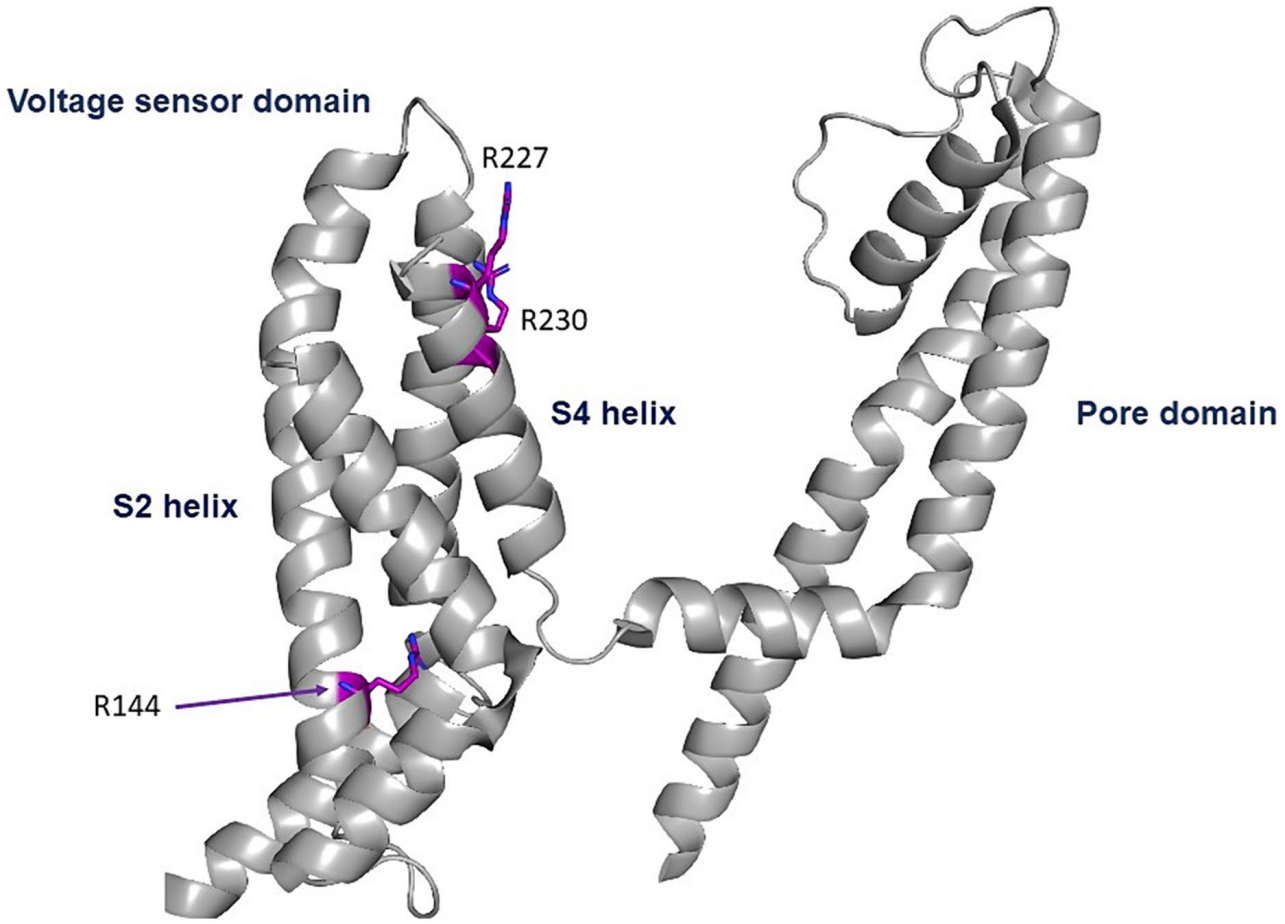
Location of the pathogenic variants. Cryo-EM structure of the voltage sensor and pore domains of the Kv7.2 subunit, using the Kv7.2 channel as a template (7CR0 template from the PDB repository). All three variants are located in the voltage sensor area. The KCNQ2 residue R144 is highly conserved among Kv channels and is located at the bottom of the S2 segment of the voltage sensor domain. The KCNQ3 residues R227 and R230 correspond to the two outermost arginine residues of the KCNQ3 S4 segment of the voltage sensor domain.

The three GoF variants correspond to *KCNQ2 (p.Arg144Gln)* and *KCNQ3 (p.Arg227Gln, p.Arg230Cys)*. The *KCNQ2* residue R144 is highly conserved among Kv channels and is located at the bottom of the S2 segment of the voltage sensor domain (Figure 1; Miceli et al., 2022). The *KCNQ3* residues R227 and R230 correspond to the two outermost arginine residues of the *KCNQ3* S4 segment of the voltage sensor domain (Figure 4; Sands et al., 2019).

In patients #1–3, the clinical phenotype was developmental encephalopathy, with intellectual disability and autistic spectrum disorder. All patients were non-verbal and were enrolled in special education programs. They never had seizures, but EEGs in patients #1 and 3 revealed multifocal spike and wave activity during sleep. The current EEG during sleep in patient #2 was normal, but in a previous EEG at 4 years of age interictal epileptiform activity was reported. Patients were referred for genetic diagnosis due to autism spectrum disorder, and a whole sequencing trio was performed as part of the routine evaluation. Three known *de novo* pathogenic variants in the *KCNQ3* gene were revealed, all previously reported as causing gain in Kv7.3 channel function: patient #1 with the p.Arg227Gln variant (Sands et al., 2019), patients #2 and #3 with the p.Arg230Cys variant (Miceli et al., 2015; Sands et al., 2019; Table 1).

In patient #4, the clinical phenotype was developmental encephalopathy with epilepsy. She presented with developmental delay and was diagnosed at 3 years of age with intellectual disability and autism; therefore, was referred to a genetic diagnosis. Whole-exome sequencing trio analysis revealed a *de novo* known variant in the *KCNQ2* gene, p.Arg144Gln, consistent with a GoF hyperactive Kv7.2 channel (Miceli et al., 2015, 2022; Table 1). At 3.5 years of age, she presented with atonic seizures and multifocal spike and wave activity during sleep on the EEG and was diagnosed with myoclonic atonic epilepsy. In an attempt to practice precision medicine, based on the pathogenic variant in the *KCNQ2* gene, she was started on the sodium channel blocker carbamazepine. This treatment caused a severe increase in the frequency of the drop attacks; therefore, it was stopped, and seizures spontaneously improved. Subsequently, she received no chronic antiseizure medication and was seizure-free, except for several days twice a year when she had recurrent atonic seizures, successfully treated with benzodiazepines.

#### 3.2.2 Treatment

All patients completed 12 months of treatment. After tapering up the drug, the therapeutic dosage was 5 mg in patients #2 and #4; at the 4-week visit, the dosage was reduced due to side effects to 2.5 mg and afterward increased to 5 mg. In patients #1 and #3, the therapeutic dosage was 2.5 mg; after 6 months, in patient #1, the dosage was increased to 5 mg, according to her age. Patients #2 and #4 reported mild side effects. Patient #2 had mild difficulty with micturition, which resolved completely with dose reduction and did not recur with an increase in dosage. Patient #4 had mild irritability, which was not influenced by dosage reduction and did not resolve. All caregivers opted to continue the therapy after the study’s completion.

### 3.3 Outcome measures

#### 3.3.1 CARS-2

**Figure 5:**
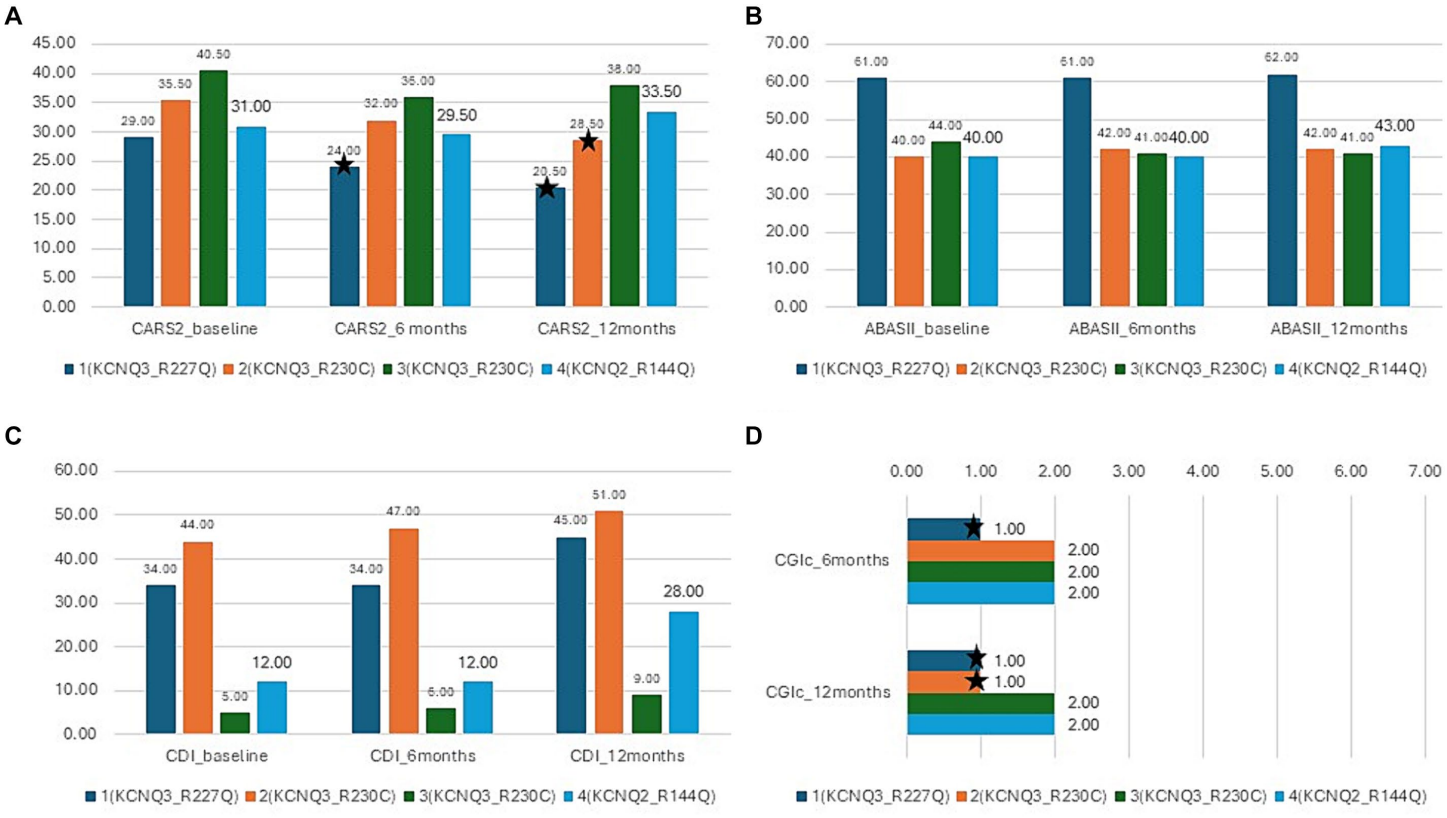
Results of the interventional study. Blue- patient 1,p.Arg227Gln, KCNQ3, orange-patient 2, p.Arg230Cys, KCNQ3, gray -patient 3, p.Arg230Cys, KCNQ3, yellow-patient 4, p.Arg144Gln, KCNQ2; **(A)** CARS-2 outcome at baseline, 6 months, and 12 months, ^*^depicts improvement over 4.5 points (clinically relevant). **(B)** ABAS-II outcome at baseline, 6 months, and 12 months. **(C)** CDI outcome at baseline, 6 months, and 12 months. **(D)** CGI-c at 6 months and 12 months.

There was a mean decrease in the CARS-2 of 3.8 ± 4.9 from 34 ± 5.1 at baseline to 30.1 ± 7.4 after 12 months (Friedman test, *p* > 0.05) (Table 2; Figure 5A). In half of the patients, the reduction was clinically relevant, over the threshold of 4.5 points improvement in CARS-2. Patient #1 had a 5-point decrease in CARS-2 at 6 months, and an 8.5-point decrease at 12 months (Table 2; Figure 5A). Patient #2 had a 3-point decrease in CARS-2 at 6 months and a 6.5-point decrease at 12 months (Table 2; Figure 5A). After treatment, both patients tested out of the screening range for autism (patient #2 borderline); patient #1 started talking fluently.

**Table 2:** Outcome measures in the interventional study.

Test	Variant	Baseline	6 months	12 months	Clinically relevant	Statistical significance
CARS-2	KCNQ3_R227Q KCNQ3_R230C KCNQ3_R230C KCNQ2_R144Q	29.00 35.50 40.50 31.00	24.00 32.00 36.00 29.50	20.50 28.50 38.00 33.50	+ +	NS
ABAS-II	KCNQ3_R227Q KCNQ3_R230C KCNQ3_R230C KCNQ2_R144Q	61.00 40.00 44.00 40.00	61.00 42.00 41.00 40.00	62.00 42.00 41.00 43.00		NS
Conceptual	KCNQ3_R227Q KCNQ3_R230C KCNQ3_R230C KCNQ2_R144Q	63.00 49.00 49.00 45.00	57.00 53.00 45.00 45.00	65.00 53.00 45.00 51.00		NS
Social	KCNQ3_R227Q KCNQ3_R230C KCNQ3_R230C KCNQ2_R144Q	65.00 53.00 50.00 48.00	65.00 53.00 48.00 48.00	80.00 55.00 48.00 60.00		NS
Practical	KCNQ3_R227Q KCNQ3_R230C KCNQ3_R230C KCNQ2_R144Q	64.00 40.00 43.00 41.00	70.00 40.00 45.00 41.00	61.00 40.00 43.00 40.00		NS
CDI	KCNQ3_R227Q KCNQ3_R230C KCNQ3_R230C KCNQ2_R144Q	34.00 44.00 5.00 12.00	34.00 47.00 6.00 12.00	45.00 51.00 9.00 28.00		*P* < 0.05
Social	KCNQ3_R227Q KCNQ3_R230C KCNQ3_R230C KCNQ2_R144Q	22.00 19.00 8.00 9.00	17.00 23.00 4.00 10.00	26.00 25.00 12.00 15.00		*p* = 0.05
Self-help	KCNQ3_R227Q KCNQ3_R230C KCNQ3_R230C KCNQ2_R144Q	18.00 18.00 8.00 11.00	17.00 19.00 8.00 12.00	23.00 22.00 8.00 15.00		NS
Gross motor	KCNQ3_R227Q KCNQ3_R230C KCNQ3_R230C KCNQ2_R144Q	22.00 13.00 6.00 6.00	23.00 13.00 4.00 7.00	26.00 14.00 10.00 10.00		*P* < 0.05
Fine motor	KCNQ3_R227Q KCNQ3_R230C KCNQ3_R230C KCNQ2_R144Q	18.00 15.00 6.00 7.00	19.00 17.00 7.00 7.00	20.00 16.00 8.00 7.00		NS
Expressive	KCNQ3_R227Q KCNQ3_R230C KCNQ3_R230C KCNQ2_R144Q	19.00 44.00 3.00 4.00	19.00 45.00 3.00 5.00	37.00 49.00 4.00 5.00		*P* < 0.05
Comprehension	KCNQ3_R227Q KCNQ3_R230C KCNQ3_R230C KCNQ2_R144Q	29.00 42.00 7.00 9.00	32.00 46.00 9.00 9.00	39.00 47.00 9.00 8.00		NS
Letters	KCNQ3_R227Q KCNQ3_R230C KCNQ3_R230C KCNQ2_R144Q	0.00 6.00 0.00 0.00	2.00 9.00 0.00 0.00	0.00 12.00 0.00 0.00		NS
Numbers	KCNQ3_R227Q KCNQ3_R230C KCNQ3_R230C KCNQ2_R144Q	5.00 9.00 0.00 0.00	4.00 11.00 0.00 0.00	6.00 11.00 0.00 0.00		NS
CGI-c	KCNQ3_R227Q KCNQ3_R230C KCNQ3_R230C KCNQ2_R144Q		1.00 2.00 2.00 2.00	1.00 1.00 2.00 2.00	+ +	ND

Clinically relevant result, over 4.5-point decrease from baseline on CARS-2 or very much improved on CGI (CGI + 1). Statistical significance- Friedman test. NS, not significant; ND, not done.

#### 3.3.2 ABAS-II

There was almost no increase in the score after 12 months (from 46.2 ± 10 to 47 ± 10) (Friedman test, *p* > 0.05) (Table 2; Figure 5B). In the social subscales, patient #1 had a 23% increase from 65 (1st percentile) to low normal 80 (9th percentile); patient number #4 had a 14% increase from 48 (0.1th percentile) to 60 (0.4th percentile) (Table 2).

#### 3.3.3 CDI

The CDI score increased by 65 ± 52.8% in 12 months (from 23.7 ± 18.3 to 33.2 ± 18.8) (Friedman test, *p* < 0.05^*^) (Table 2; Figure 5C). Specific subtests that showed statistically significant improvement were social, gross motor, and expressive language (Table 2). In patient #1, there was a 32% improvement from 34 to 45, with the expressive language subtest improving from 19 to 37 (94% increase). Patient #4 had a 133% increase in the score, from 12 to 28, with no specific subtests showing better results.

#### 3.3.4 CGI-c and general improvement

All caregivers reported improvement in disease symptoms, as graded by the CGI-c score. In patients #1 and #2, the improvement was prominent (CGI-c = 1, very much improved) (after 6 months, the grade was 2 in patient #2) (Figure 5D). In patients #3 and #4, the improvement was moderate (CGI-c = 2, moderately improved). In addition, caregivers mentioned specific areas of improvement: attention span, participation, expressive language, and understanding.

## 4 Discussion

*KCNQ2*-linked pathogenic variants include a range of overlapping neonatal epileptic phenotypes ranging from mild self-limited familial neonatal epilepsy to severe neonatal-onset developmental and epileptic encephalopathy (Miceli et al., 1993; Biervert et al., 1998; Singh et al., 2003; Weckhuysen et al., 2012). Additional, less frequent phenotypes consisting of neonatal encephalopathy with non-epileptic myoclonus, infantile or childhood-onset developmental and epileptic encephalopathy, as well as isolated intellectual disability without epilepsy, have also been reported (Miceli et al., 1993). *KCNQ3*-related pathogenic variants comprise self-limited familial neonatal epilepsy and self-limited familial infantile epilepsy, seizure disorders that occur in children who typically have normal psychomotor development (Miceli et al., 1993; Singh et al., 2003). Additional *KCNQ3*-related neurodevelopmental disorders with and without epilepsy have also been described (Miceli et al., 1993). GoF variants in *KCNQ2* and *KCNQ3* genes were recently reported to cause profound developmental delay, neonatal non-epileptic myoclonus, and later-onset epilepsy such as infantile spasms or the autism phenotype with no clinical seizures, respectively (Miceli et al., 2015; Millichap et al., 2017; Mulkey et al., 2017; Sands et al., 2019; Miceli et al., 2022). These patients are not currently treated with appropriate personalized medicine, since Kv7.2 channel blockers are not available. The tricyclic antidepressant amitriptyline was shown to have a direct inhibitory effect on Kv7.2 and Kv7.3 channels at toxic levels (Punke and Friederich, 2007). Patch clamp analysis studies by Miceli et al. (2022) and Bayat et al. (2023) showed that amitriptyline (1 and 10 μM) dose-dependently and reversibly inhibited GoF Kv7.2 channels caused by the p.Arg144Gln (Miceli et al., 2022), p.Tyr141Asn, and p.Gly239Ser (Bayat et al., 2023) variants. In addition, Bayat et al. (2023) showed recently a beneficial effect of 1 mg/kg amitriptyline on cognitive functions in an 8-year-old patient bearing the p.Gly239Ser variant.

In the present study, we evaluated the therapeutic potential of donepezil, a reversible acetylcholinesterase inhibitor approved by the FDA (Bryson and Benfield, 1997), in four children affected by GoF variants of *KCNQ2* and *KCNQ3* encephalopathy. Donepezil is the most commonly prescribed cholinesterase inhibitor for treating Alzheimer’s disease, which reversibly inactivates acetylcholinesterase, thereby inhibiting the hydrolysis of acetylcholine and increasing its concentration in the synaptic space of cholinergic neurons (Bryson and Benfield, 1997). In a randomized study in children with autism, aged 5–18 years (Gabis et al., 2019), showed that 3-month treatment with donepezil hydrochloride and choline supplement produces a sustainable beneficial effect (6 months) on receptive language skills in children, especially in those younger than 10 years of age. Improvement was attributed to a non-specific mechanism in autism, related to increased levels of synaptic acetylcholine. The drug was well tolerated, with mild side effects mainly observed in those over 10 years of age: behavior worsening, agitation, anxiety, sleep disturbance, skin rash, gastrointestinal disturbance, tremor, headache, and urination (Gabis et al., 2019). Cardiac toxicity, which was rarely reported in geriatric patients on polytherapy, was not present in children (Gabis et al., 2019).

Here, we found that donepezil treatment produced an improvement in cognitive skills in four children with GoF variants in *KCNQ2* and *KCNQ3*. Autistic features improved, as shown by a decrease in the CARS-2 score (Table 2; Figure 5A). As expected in such a small sample size, the improvement was not statistically significant. However, in two patients with *KCNQ3* variants, the improvement was over the 4.5-point threshold suggested in the literature as being clinically relevant (Table 2; Figure 5A). In these two patients, the clinical improvement graded by the CGI score was prominent (Table 2; Figure 5D), and the CARS-2 scale tested out of the range for autism. Expressive language also improved after treatment (Table 2), especially in a 3.5-year-old girl bearing the p.Arg227Gln variant who started speaking fluently. While we cannot rule out some spontaneous improvement in speech at this age, the improvement in communication skills, as demonstrated by the CARS-2 score within the normal range, suggests that the effect is drug-related rather than age-related. Additional domains showing improvement were social skills and gross motor skills (Table 2). Caregivers mentioned also improved attention span, but the change was not evident in the outcome measures, indicating that the scales were not sensitive enough.

We have no explanation for the different magnitudes of responses in patients or predictors for a positive response. Interestingly, we had two patients who had the same variant in the KCNQ3 gene (p.Arg230Cys). We would have expected the younger 2.5-year-old girl to have a better response, but perplexingly, the results showed improvement in the 8-year-old boy. Differences in the genetic background, such as modifier genes or epigenetic differences, could account for this variability. Since none of the patients were on other medications while receiving donepezil, concomitant therapy could not influence the outcome. It should be noted that non-compliance cannot be ruled out, since we did not check for treatment adherence.

Our outcome measures were focused on cognitive functions, since three patients with *KCNQ3* variants had no seizures. The patient bearing the p.Arg144Gln *KCNQ2* variant had epilepsy, which deteriorated when treated with the sodium channel blocker carbamazepine, suited for LoF variants. The mechanism of epilepsy in the KCNQ2 GoF phenotype is unclear, since these variants should lead to excessive inhibition of neuronal firing and hence a lesser tendency to seizures. However, paradoxical neuronal hyperexcitability and seizures could be due to a predominant GoF in inhibitory neurons by shifting the Kv7.2 activation gating to hyperpolarized potentials (Millichap et al., 2017), or by stabilizing the Kv7.2 channel open states (Miceli et al., 2015). Treatment with a sodium channel blocker in our patient might have diminished the availability of sodium channels in the inhibitory neurons, causing seizure aggravation, similar to Dravet syndrome. Treatment with donepezil did not cause seizure aggravation, stressing the importance of precision medicine. The therapeutic potential of donepezil may also be of great value in children with epilepsy caused by GoF variants in *KCNQ2*, since its administration was recently shown to confer robust protection against induced seizures in a mouse model of Dravet syndrome (Wong et al., 2019).

Importantly, we showed that the cholinergic drug donepezil acts as an indirect Kv7.2/3 channel blocker in hippocampal neurons (Figure 1). Donepezil produced a significant inhibition of the M-current density in excitatory cultured hippocampal neurons (67%) and, to a lesser extent, that of inhibitory neurons (59%) (Figures 1, 2). We consistently found that the M-current density in inhibitory neurons was slightly lower than that of excitatory neurons. Thus, donepezil has a lower level of M-current to inhibit, which may account for the weaker impact of donepezil on inhibitory neurons. It is well known that muscarinic receptor signaling inhibits the voltage-dependent potassium M-current, which regulates neuronal excitability (Delmas and Brown, 2005). M1and M3 muscarinic receptors, which are coupled to Gq/11, stimulate phospholipase C (PLC-β), resulting in the degradation of PIP2 to diacylglycerol (DAG) and inositol-1,4,5-triphosphate (IP3), leading to calcium release and protein kinase C (PKC) activation (Delmas and Brown, 2005; Kosenko et al., 2012). Our results of the preventing atropine effect of donepezil action on the M-current and on the hippocampal network firing clearly confirm the involvement of muscarinic receptor signaling. The depression of the M-current density by donepezil could arise from three main mechanisms. One explanation would be the depletion of PIP2 resulting from its breakdown by PLC-β. Indeed, the direct interaction of PIP2 with the Kv7.2/3 channels is essential to stabilizing the gating open state (Delmas and Brown, 2005; Gamper and Shapiro, 2007a). Another mechanism would be that the DAG resulting from PIP2 breakdown by PLC-β can directly activate PKC, which was previously shown to mediate muscarinic receptor-mediated inhibition of Kv7.2/3 channel activity (Nakajo and Kubo, 2005; Surti et al., 2005). Interestingly, a recent study reported that the systemic administration of donepezil induces the phosphorylation of Kv7.2 in the nucleus accumbens of mice at threonine 217 (T217) via PKC activation for aversive learning (Faruk et al., 2022). A muscarinic M1 receptor antagonist suppressed this phosphorylation effect of donepezil (Faruk et al., 2022). Thus, the phosphorylation of Kv7.2 by donepezil at T217 appears to reduce channel activity (Surti et al., 2005). Threonine 217 is located at the intracellular linker S4-S5, a region known to interact with PIP2 (Zhang et al., 2013). Thus, it is possible that adding a negative charge by phosphorylation at T217 could prevent the interaction of the channel with PIP2 and therefore trigger the inhibition of the M-current (Faruk et al., 2022). A third putative mechanism is the Ca^2+^−mediated inhibition of the M-current (Marrion, 1997; Cooper, 2012). Indeed, the PIP2 breakdown by PLC-β not only generates DAG but also IP3, which will release Ca^2+^ from intracellular ER stores, thereby increasing cytoplasmic Ca^2+^ levels and inhibiting the M-current. Overall, the decreased M-current density is expected to have an impact on hippocampal network excitability. In line with this assumption, we found that donepezil potently increased the spontaneous firing of excitatory hippocampal neurons. Moreover, atropine co-application not only prevented the hyperexcitability triggered by donepezil but even reduced the firing frequency, suggesting the existence of a tonic cholinergic excitability in the cultured hippocampal network we used in this study. How does the GoF of KCNQ2/3 mutations affect network excitability? Although it is extremely difficult to predict the outcome of the GoF of K^+^ channels at the whole neural network level, an increased function of subthreshold K^+^ channels such as the M-current was suggested to over-activate the hyperpolarization-activated non-selective cation current Ih (Robinson and Siegelbaum, 2003), resulting in secondary depolarizations. Computational modeling experiments suggest that an increased function of M-current is unable to increase principal neuron excitability by Na^+^ channels repriming because the increase in Na^+^ channels availability is not sufficient to counterbalance the mutation-induced hyperpolarization (Miceli et al., 2015); instead, because of the larger input resistance observed in interneurons compared with principal neurons, a larger M-current would preferentially affect the inhibitory interneuron, thereby leading to increased excitability of the principal neuron. Illustrating the often unpredictable and complex outcome of KCNQ2/3 GoF mutations, a recent study showed that the KCNQ2 GoF mutant (R201C) depresses the firing activity of retrotrapezoid nucleus neurons (RTNs) (Soto-Perez et al., 2023). Kcnq2R201C/+ GOF mice breathe normally under baseline conditions but show a blunted ventilatory response to CO2 preferentially during the light/inactive state, when the inhibition of Kcnq2 channels in RTN neurons by wake-on neurotransmitters is expected to be minimal, thereby decreasing the central chemoreflex (Soto-Perez et al., 2023). In any case, donepezil is likely predicted to prevent the consequences of KCNQ2/3 GoF mutations.

In conclusion, our laboratory data support a specific effect of donepezil in treating patients with GoF variants in the *KCNQ2* and *KCNQ3* genes. The subsequent open-label interventional study raised the need to repurpose donepezil as a personalized approach for these patients. Future studies should also explore the effect of choline supplementation on the magnitude of the response, as well as the repurposing of other muscarine receptor antagonists, which should show a similar effect. This study emphasizes the importance of small-scale translational studies, born at the interface between basic and clinical science, as a cornerstone in implementing personalized medicine. Further large-scale placebo-controlled studies are needed to confirm our results.

## Data availability statement

The raw data supporting the conclusions of this article will be made available by the authors, without undue reservation.

## Ethics statement

The studies involving humans were approved by the Wolfson Medical Center Ethical Committee IRB#0150-23-WOMC. The studies were conducted in accordance with the local legislation and institutional requirements. Written informed consent for participation in this study was provided by the participants' legal guardians/next of kin. Written informed consent was obtained from the minor(s)' legal guardian/next of kin for the publication of any potentially identifiable images or data included in this article.

## Author contributions

AN: Conceptualization, Data curation, Formal analysis, Investigation, Methodology, Project administration, Visualization, Writing – original draft, Writing – review & editing. LB: Funding acquisition, Investigation, Writing – review & editing. AB-B: Investigation, Writing – review & editing. LR: Investigation, Writing – review & editing, Conceptualization. HA: Investigation, Writing – review & editing. RS: Writing – review & editing, Investigation. LG: Conceptualization, Writing – review & editing. BA: Conceptualization, Funding acquisition, Investigation, Methodology, Supervision, Visualization, Writing – original draft, Writing – review & editing.

## Footnotes

^1^http://www.ilae.org/Visitors/News/documents/GSK_Retigabine_market_withdrawal.pdf
